# Metabolic Risk Profile and Graft Function Deterioration 2 Years After Kidney Transplant

**DOI:** 10.1001/jamanetworkopen.2023.49538

**Published:** 2023-12-27

**Authors:** Jiayi Yan, Xiaoqian Yang, Jieying Wang, Hong Cai, Xiajing Che, Liang Ying, Tianyi Zhang, Qian Chen, Jia Xia, Leyi Gu, Xiaodong Yuan, Ruoyang Chen, Dawei Li, Zhihong Liu, Kun Dong, Long He, Ming Zhang, Shan Mou

**Affiliations:** 1 Department of Nephrology, Molecular Cell Lab for Kidney Disease, Shanghai Peritoneal Dialysis Research Center, Ren Ji Hospital, Uremia Diagnosis and Treatment Center, Shanghai Jiao Tong University School of Medicine, Shanghai, China; 2 Academy of Integrative Medicine, Shanghai University of Traditional Chinese Medicine, Shanghai, China; 3 Clinical Research Center, Ren Ji Hospital, Shanghai Jiao Tong University School of Medicine, Shanghai, China; 4 Department of Urology, Ren Ji Hospital, Shanghai Jiao Tong University School of Medicine, Shanghai, China; 5 Department of Urology, Shanghai General Hospital, Shanghai Jiao Tong University School of Medicine, Shanghai, China; 6 Organ Transplantation Center, The First Affiliated Hospital of Guangxi Medical University, Nanning, China; 7 Organ Transplantation Center, General Hospital of Northern Theater Command, Shenyang, China

## Abstract

**Importance:**

Studies exploring the association of body weight and metabolic status with graft function deterioration (GFD) after kidney transplantation have produced inconsistent findings. Few studies have examined whether metabolically healthy overweight or obesity (MHO) may contribute to GFD.

**Objective:**

To evaluate associations of overweight or obesity and metabolic disorders with GFD in recipients of kidney transplant.

**Design, Setting, and Participants:**

This multicenter retrospective cohort study was conducted from January 1, 2020, through June 30, 2021, with a follow-up period of 2 years after kidney transplantation. Participants included adult recipients of cadaveric kidney transplant in 4 transplantation centers in China. Participants were classified as 4 metabolic phenotypes according to their BMI and metabolic status. Data were analyzed from July to August 2023.

**Exposures:**

Overweight and obesity were characterized by body mass index (BMI; calculated as weight in kilograms divided by height in meters squared) of 24 or greater. Metabolic disorder was identified by existence of a minimum of 2 of 4 conditions: hypertension, hyperglycemia, increased triglyceride, and decreased high-density lipoprotein cholesterol.

**Main Outcome and Measures:**

The main outcome was GFD, defined as a decrease in estimated glomerular filtration rate of at least 25% within 6 months to 2 years after transplant.

**Results:**

A total of 1260 adult recipients of cadaveric kidney transplant (mean [SD] age, 43.97 [11.51] years; 755 [59.92%] male) were included in the study, and 127 (10.08%) participants experienced the primary outcome of GFD during follow-up. After accounting for confounding factors in multivariable analyses, overweight or obesity (odds ratio [OR], 1.64; 95% CI, 1.10-2.44; *P* = .02) and metabolic disorder (OR, 1.71; 95% CI, 1.12-2.63; *P* = .01) were associated with increased risk of GFD. The MHO subgroup exhibited a greater risk for GFD (OR, 2.37; 95% CI, 1.01-5.57; *P* = .048) compared with participants who did not have overweight or obesity or metabolic disorder. All components of metabolic disorder, with the exception of elevated triglyceride, were associated with GFD. There was a dose-response association of number of metabolic disorder components (OR per 1 additional condition, 1.40; 95% CI, 1.20-1.63; *P* < .001) and BMI (OR per 1-unit increase, 1.90; 95% CI, 1.03-1.15; *P* = .002) with increased risk for GFD. A nonlinear association was observed between BMI and risk of GFD.

**Conclusions and Relevance:**

In this cohort study of recipients of cadaveric kidney transplant, individuals with overweight or obesity or metabolic disorder had a significantly higher risk of experiencing GFD. Individuals with MHO had an elevated risk for graft function deterioration. Additional studies with larger sample size and longer follow-up are necessary to validate our findings.

## Key Points

**Question:**

Are body mass index and metabolic conditions associated with graft function deterioration in recipients of kidney transplant?

**Findings:**

In this cohort study of 1260 recipients of kidney transplant in China, both body mass index and metabolic conditions early after kidney transplantation were significantly associated with an increased risk of graft function deterioration in the 2 years after transplant.

**Meaning:**

These findings suggest that it is important to regularly monitor patient metabolic risk profile in the early stage after kidney transplantation.

## Teaser

This cohort study evaluates associations of overweight or obesity and metabolic disorders with graft function decline in adult recipients of kidney transplant in China.

## Introduction

Metabolic syndrome encompasses a cluster of interconnected cardiometabolic risk factors, including overweight or obesity, high blood pressure, dyslipidemia, and alterations in glucose metabolism, with insulin resistance proposed as the mutual pathogenic background. It is widely recognized that metabolic syndrome is a critical public health concern and major cause of cardiovascular incidents and mortality.^1,2,3,4^ Numerous studies have proposed an association of metabolic syndrome with the onset of chronic kidney disease (CKD). Additionally, among individuals already diagnosed with CKD, metabolic syndrome may accelerate the decline in glomerular filtration rate (GFR).^5,6,7,8^ Overweight and obesity are key components of metabolic syndrome. The presence of metabolic syndrome is often associated with overweight or obesity.^9,10^ However, there exists a paradoxical association of overweight and obesity with CKD progression of the native kidney.^11,12,13^

Previous investigations have identified a subgroup of individuals with overweight or obesity with metabolically healthy overweight or obesity (MHO). The unique phenotype exhibits distinctive traits of reduced metabolic burden, including better-controlled blood pressure and glucose, diminished insulin resistance, and improved lipid and inflammatory characteristics, compared with conventional overweight or obesity.^13,14^ The existence of healthy overweight or obesity and its representation of a benign condition remains a contentious topic.^15,16,17^ Remarkably, individuals with MHO have been found to present an elevated risk of developing CKD.^18,19^ This finding propelled our assumption that overweight and obesity could have a detrimental effect on renal outcomes, and metabolic irregularities could potentially influence the association of overweight or obesity with kidney homeostasis.

Scarce information exists regarding how body weight and metabolic status may influence the risk for graft outcomes for recipients of kidney transplant, a population with escalated risk for metabolic disturbances.^20,21^ Use of immunosuppressants is associated with an increase in the prevalence and severity of cardiometabolic irregularities.^22^ Improving long-term allograft survival remains a challenge. Patient death, often related to cardiovascular issues, and chronic allograft dysfunction (CAD) are predominant causes of late graft failure in recipients of kidney transplant. CAD is believed to be the result of complex, ongoing response-to-injury pathophysiological damage involving both immunological and nonimmunological factors. Overweight or obesity, hypertension, hyperglycemia, and dyslipidemia are nonimmunological factors, and they are primarily associated with CAD after the first year.^23^ The intersection of these constituent components has led to a unified hypothesis of an association of metabolic disorders with CAD.^24^ However, there are inconsistent results about the associations of metabolic status with CAD development, and few studies have looked into metabolic subtypes.^21,25,26,27,28^ Hence, the objective of this study was to decipher the associations among overweight or obesity, metabolic disorders, and the decline of graft function in recipients of kidney transplant through data analyses involving a large sample size and multiple centers. We are particularly interested in studying the outcome of kidney graft in individuals with the MHO phenotype. It is crucial to distinguish individuals who are at an elevated risk of experiencing unfavorable outcomes at an early stage for appropriate risk classification, which may facilitate preventive strategies against graft failure.

## Methods

This cohort study was approved by the ethics committees of all participating hospitals. Informed consent was obtained from all study participants. The study was conducted in accordance with the principles of the Declaration of Helsinki. This study followed the Strengthening the Reporting of Observational Studies in Epidemiology (STROBE) reporting guideline.

### Study Participants

This study is a retrospective analysis of the registry database of a prospectively established cohort, the Construction of Solid Organ Transplantation Database and Biobank. Additional information on the project is provided in the eMethods in Supplement 1. In this study, we included all adult recipients of cadaveric kidney transplant whose transplantations were conducted from January 1, 2020, through June 30, 2021, in 4 transplantation centers and who survived with a functioning allograft beyond the first 6 months after transplantation (baseline was considered 6 months after transplant). Exclusion criteria were apparent systemic illnesses, such as malignant neoplasm, severe heart failure, or cirrhosis; pregnancy; multiorgan transplantation; and retransplantation. During the study, 1435 cadaveric kidney transplantations were conducted in participating centers, of which 1260 recipients of transplant met the eligibility criteria. The evaluation process for eligibility is delineated in eFigure 1 in Supplement 1. We extracted relevant clinical information from our registry database regarding the recipient, donor, and transplant characteristics.

### Definitions of Weight and Metabolic Status

Overweight or obesity in this context refers to individuals with a body mass index (BMI; calculated as weight in kilograms divided by height in meters squared) equal to or greater than 24.0, per the weight criteria outlined by the Working Group on Obesity in China.^29^ To define metabolic disorder, we focused on 4 clinical and laboratory parameters: hypertension, low high-density lipoprotein cholesterol (HDL-C), hypertriglyceridemia, and hyperglycemia. The specific definition for metabolic components and detailed considerations are provided in the eMethods in Supplement 1. Patients were categorized into 4 phenotypes according to the baseline assessment: metabolically healthy without overweight or obesity (MHNO), MHO, metabolically unhealthy without overweight or obesity (MUNO), and metabolically unhealthy overweight or obesity (MUO).

### Outcomes

We defined the primary outcome, graft function deterioration (GFD), as decline in estimated GFR (eGFR) equal to or greater than 25% between 6 months and 2 years after transplant. For patients who experienced graft loss (death or graft failure) before 2 years, the last eGFR level was used for analysis. Detailed considerations are provided in the eMethods in Supplement 1.

### Immunosuppression

The immunosuppression protocol is provided in the eMethods in Supplement 1. Patients underwent routine assessments of levels of immunosuppressants to attain optimal therapeutic concentrations.

### Statistical Analysis

Continuous variables were expressed as mean (SD) or median (IQR), while categorical variables were presented as counts with percentages. We used *t* test or analysis of variance test to compare continuous variables, and the Shapiro-Wilk test was used to assess the normality of the data. Categorical variables were compared using χ^2^ or Fisher exact tests. The Kruskal-Wallis test was used for data with skewed distribution. Multivariable logistic regression models were used to estimate odds ratios (ORs) and 95% CIs for GFD. Model 1 was adjusted for age, sex, and eGFR. Model 2 was adjusted for model 1 variables plus panel reactive antibody, hemoglobin, albumin, and primary disease. Model 3 was created after additional adjustment for donor parameters, such as donor age, creatinine, BMI, and human leucocyte antigen mismatch, in addition to the factors included in model 2. To estimate the possible nonlinear associations between BMI and GFD, we used a restricted cubic splines model with 4 knots at the 5th, 35th, 65th, and 95th percentiles. The spline model adjusted for age, sex, primary disease, and other metabolic components. We tested for potential nonlinearity with a likelihood ratio test. The rcssci package in R statistical software version 3.5.0 (R Project for Statistical Computing) was used to visualize splines. We conducted 3 sensitivity analyses. First, we excluded individuals with diabetes at transplant. Second, we excluded individuals with hypertensive nephrosclerosis or diabetic kidney disease as the primary disease. Hypertensive nephrosclerosis and diabetics kidney disease are the 2 most common secondary kidney diseases resulting from metabolic disorders, and they may have direct detrimental impact on the kidney graft after transplantation. Third, we reanalyzed our findings using 20% and 30% cutoff values for eGFR deterioration. Generalized estimating equation analysis was used to evaluate the association of metabolic subtypes with changes in eGFR at different time points during the follow-up. The dependent variable was GFD, defined as a binary outcome variable of equal to or greater than 25% decline in eGFR from 6 months after transplant or not. eGFR was assessed at 12, 18, and 24 months after transplant in the model. Kaplan-Meier analysis of eGFR decline and graft loss was also conducted. Data were complete for eGFR values and missing for less than 10% of observations for all other variables. Missing data were handled by multiple imputation using multivariate imputation by chained equations. We used SPSS software version 26 (IBM) and R statistical software version 3.5.0 for statistical analyses. A 2-sided *P* < .05 was considered statistically significant. Data were analyzed from July to August 2023.

## Results

### Baseline Characteristics

Of the 1260 adult recipients of cadaveric kidney transplant included, 755 (59.92%) were male, and the mean (SD) age was 43.97 (11.51) years. Patients were classified according to BMI and metabolic conditions. There were 354 individuals (28.10%) classified as overweight or obese, and 811 individuals (64.37%) had metabolic disorder. Individuals who were overweight or obese or had any metabolic disorder were generally older and predominantly male (Table 1).

**Table 1.** Baseline Characteristics of Patients Categorized by Body Weight and Metabolic Status

Characteristic	Participants, No. (%)			*P* value	Participants, No. (%)		*P* value
	All (N = 1260)	Overweight or obese^a^			Metabolic disorder		
		Yes (n = 354)	No (n = 906)		Yes (n = 811)	No (n = 449)	
Age, mean (SD), y	43.97 (11.51)	46.49 (11.08)	42.99 (11.53)	<.001	45.43 (11.34)	41.31 (11.36)	<.001
Sex							
Male	755 (59.92)	260 (73.45)	495 (54.64)	<.001	508 (62.48)	247 (55.26)	.02
Female	505 (40.08)	94 (26.55)	411 (45.36)		305 (37.52)	200 (44.74)	
BMI	22.04 (3.45)	26.26 (2.15)	20.39 (2.26)	<.001	22.57 (3.49)	21.08 (3.16)	<.001
PRA positive	90 (7.14)	25 (7.06)	65 (7.17)	.96	64 (7.87)	26 (5.82)	.22
Time receiving dialysis, median (IQR), mo	14.00 (6.00-34.00)	16.00 (6.00-31.00)	13.00 (5.00-35.00)	.27	15.00 (6.00-35.00)	13.00 (5.00-32.00)	.25
Diabetes at transplant	326 (25.87)	133 (37.57)	193 (21.30)	<.001	310 (38.13)	16 (3.58)	<.001
Primary disease							
Glomerulonephritis	976 (77.46)	260 (73.45)	716 (79.03)	.004	625 (76.88)	351 (78.52)	<.001
ADPKD	59 (4.68)	19 (5.37)	40 (4.42)		34 (4.18)	25 (5.59)	
Metabolic (hypertension, DKD)	88 (6.98)	39 (11.02)	49 (5.41)		74 (9.10)	14 (3.13)	
Other	137 (10.87)	36 (10.17)	101 (11.15)		80 (9.84)	57 (12.75)	
Donor							
Age, mean (SD), y	46.71 (16.16)	47.64 (14.72)	46.35 (16.68)	.20	46.83 (16.39)	46.49 (15.75)	.72
BMI, mean (SD)	22.92 (3.42)	23.16 (3.34)	22.82 (3.45)	.12	23.00 (3.54)	22.75 (3.18)	.21
Creatinine, mean (SD), mg/dL	0.90 (0.64-1.18)	0.93 (0.68-1.19)	0.89 (0.63-1.18)	.11	0.90 (0.66-1.19)	0.89 (0.61-1.13)	.15
Ischemic time, mean (SD), h	20.56 (5.41)	20.45 (5.09)	20.09 (4.98)	.19	20.65 (5.29)	20.19 (5.12)	.22
HLA mismatch, mean (SD)	3.03 (1.64)	3.04 (1.70)	3.02 (1.62)	.86	3.05 (1.62)	2.98 (1.68)	.46
Immunosuppressants							
Tacrolimus	1063 (84.37)	300 (84.75)	763 (84.22)	.74	690 (85.08)	373 (83.07)	.38
Cyclosporin	190 (15.08)	52 (14.69)	138 (15.23)		120 (14.80)	70 (15.59)	
Rapamycin	7 (0.56)	2 (0.56)	5 (0.55)		1 (0.12)	6 (1.34)	
Tacrolimus level, mean (SD), ng/mL	6.37 (1.68)	6.26 (1.77)	6.50 (1.82)	.311	6.21 (1.71)	6.52 (1.93)	.24
Laboratory values, mean (SD)							
White blood cells, 10^3^/μL	6.55 (2.82)	6.21 (2.37)	6.69 (2.97)	.008	6.50 (2.87)	6.65 (2.72)	.38
Hemoglobin, g/dL	10.26 (1.97)	10.50 (1.86)	10.17 (2.01)	.007	10.28 (1.97)	10.23 (1.99)	.68
Platelets, 10^3^/µL	210.00 (170.50-265.00)	209.00 (171.50-255.50)	212.00 (170.00-268.00)	.61	207.00 (168.00-266.00)	215.00 (174.00-263.00)	.25
Albumin, g/dL	4.20 (0.43)	4.23 (0.41)	4.19 (0.44)	.15	4.21 (0.43)	4.18 (0.44)	.21
Globulin, g/dL	2.47 (0.43)	2.48 (0.41)	2.47 (0.43)	.70	2.47 (0.43)	2.48 (0.42)	.76
ALT, U/L, median (IQR)	16.00 (11.00-25.00)	17.00 (11.25-24.00)	16.00 (11.00-25.00)	.93	16.00 (11.00-25.00)	16.00 (10.00-26.00)	.68
AST, U/L, median (IQR)	14.00 (12.00-19.00)	14.00 (11.00-18.00)	15.00 (12.00-20.00)	.001	14.00 (11.00-19.00)	15.00 (12.00-19.00)	.06
BUN, mg/dL	27.9 (13.7)	28.49 (12.13)	27.68 (14.29)	.34	28.18 (13.22)	27.37 (14.62)	.33
Creatinine, mg/dL	1.32 (1.06-1.67)	1.43 (1.15-1.75)	1.29 (1.03-1.63)	<.001	1.35 (1.08-1.67)	1.29 (1.03-1.62)	.049
eGFR, mL/min/1.73 m^2^	62.99 (22.04)	59.72 (21.34)	64.24 (22.18)	.002	61.66 (21.95)	65.54 (22.00)	.004
UA, mg/dL, median (IQR)	5.63 (4.74-6.59)	5.66 (4.82-6.72)	5.60 (4.71-6.55)	.24	5.63 (4.73-6.62)	5.58 (4.74-6.54)	.59
TG, mg/dL	1.81 (0.98)	2.04 (1.06)	1.72 (0.94)	<.001	2.06 (1.05)	1.33 (0.60)	<.001
TC, mg/dL	5.06 (1.17)	5.08 (1.17)	5.05 (1.17)	.65	5.16 (1.18)	4.86 (1.12)	<.001
HDL-C, mg/dL	1.31 (0.37)	1.24 (0.37)	1.37 (0.37)	.24	1.24 (0.37)	1.47 (0.33)	.05
FBG, mg/dL	102.16 (31.17)	106.13 (28.65)	100.72 (31.89)	.009	107.39 (36.22)	92.43 (13.51)	<.001

Abbreviations: ADPKD, autosomal dominant polycystic kidney disease; ALT, alanine aminotransferase; AST, aspartate aminotransferase; BMI, body mass index (calculated as weight in kilograms divided by height in meters squared); BUN, blood urea nitrogen; DKD, diabetic kidney disease; eGFR, estimated glomerular filtration rate; FBG, fasting blood glucose; HDL-C, high-density lipoprotein cholesterol; HLA, human leucocyte antigen; PRA, panel reactive antibody; TC, total cholesterol; TG, triglyceride; UA, uric acid.

SI conversion factors: To convert albumin to grams per liter, multiply by 10; ALT and AST to microkatals per liter, multiply by 0.0167; BUN to millimoles per liter, multiply by 0.357; creatinine to micromoles per liter, multiply by 88.4; FBG to millimoles per liter, multiply by 0.0555; hemoglobin and white blood cells to 10^9^/L, multiply by 1; TG to millimoles per liter, multiply by 0.0113; TC and HDL-C to millimoles per liter, multiply by 0.0259; UA to millimoles per liter, multiply by 0.0595.

^a^ Overweight or obese was defined as BMI greater than 24.

### Weight, Metabolic Status, and the Risk of GFD

During the follow-up period, 23 patients had graft loss, of whom 5 patients also experienced the primary GFD outcome. GFD occurred in 80 individuals not classified as overweight or obese (8.83%) and 47 individuals classified as overweight or obese (13.28%). After adjustment for confounding factors in multivariable analysis, overweight or obesity was significantly associated with an increased risk of GFD compared with weight within reference range (OR, 1.64; 95% CI, 1.10-2.44; *P* = .02). Of participants with metabolic disorder, 95 (11.43%) experienced GFD, whereas only 32 participants (7.13%) without metabolic disorder experienced GFD. After adjusting for various factors in multivariable models, metabolic disorder was significantly associated with an increased risk of GFD (OR, 1.71; 95% CI, 1.12-2.63; *P* = .01) (eTable 1 in Supplement 1).

### Risk for Adverse Outcomes Based on Metabolic Phenotypes

We compared clinical characteristics among the 4 metabolic phenotype subgroups, including 363 participants (28.81%) in the MHNO subgroup, 543 participants (43.10%) in the MUNO subgroup, 84 participants (6.67%) in the MHO subgroup, and 270 participants (21.43%) in the MUO subgroup. Compared with the other 3 subgroups, participants in the MHNO subgroup were relatively younger and more likely to be men (Table 2). During the follow-up period, GFD occurred in 22 patients in the MHNO subgroup (6.06%), 58 patients in the MUNO subgroup (10.68%), 9 patients in the MHO subgroup (10.71%), and 38 patients in the MUO subgroup (14.07%). The eGFR decline plot is shown in eFigure 2 in Supplement 1. After adjusting for confounders, the MUNO subgroup experienced 1.91-fold increased risk of GFD (OR, 1.91; 95% CI, 1.11-3.31; *P* = .02) and the MUO subgroup had 2.76-fold increased risk (OR, 2.76, 95% CI, 1.50-5.06; *P* = .001), compared with the MHNO subgroup. Interestingly, the risk for GFD was also higher in the MHO subtype compared with the MHNO subtype (OR, 2.37; 95% CI, 1.01-5.57; *P* = .048) in the fully adjusted model (Table 3). When defining the composite outcome as eGFR decline or graft loss, results were similar, except that the MHO subgroup did not exhibit a higher risk for adverse outcomes compared with the MHNO subgroup (eTable 2 in Supplement 1). Generalized estimating equation analysis showed that the MUNO, MHO, and MUO subtypes were all associated with increased odds of GFD compared with the MHNO subtype in the fully adjusted model (eTable 3 in Supplement 1). Adverse graft events of eGFR decline were also significantly higher in the MUNO, MHO, and MUO subgroups than the MHNO subgroup, while there was no significant difference in graft loss among subgroups (eFigure 3 and eFigure 4 in Supplement 1).

**Table 2.** Characteristics of Metabolic Phenotypes Categorized by Overweight or Obesity and Metabolic Disorder

Characteristic	Participant metabolic phenotype, No. (%)				*P* value
	MUO (n = 270)	MHO (n = 84)	MUNO (n = 543)	MHNO (n = 363)	
**At baseline**					
Age, mean (SD) y	46.97 (11.04)	44.94 (11.12)	44.67 (11.41)	40.47 (11.27)	<.001
Sex					
Male	196 (72.59)	64 (76.19)	312 (57.46)	183 (50.41)	<.001
Female	74 (27.41)	20 (23.81)	231 (42.54)	180 (49.59)	
BMI, mean (SD)	26.40 (2.19)	25.79 (1.93)	20.66 (2.22)	19.99 (2.26)	<.001
PRA positive	20 (7.41)	5 (5.95)	44 (8.10)	21 (5.79)	.58
Time receiving dialysis, mean (SD), mo	16.00 (6.00-31.00)	15.00 (5.25-30.75)	14.00 (6.00-36.00)	12.50 (5.00-32.00)	.52
Diabetes at transplant	130 (48.15)	3 (3.57)	180 (33.15)	13 (3.58)	<.001
Primary disease					
Glomerulonephritis	204 (75.56)	56 (66.67)	421 (77.53)	295 (81.27)	<.001
ADPKD	13 (4.81)	6 (7.14)	21 (3.87)	19 (5.23)	
Metabolic (hypertension, DKD)	32 (11.85)	7 (8.33)	42 (7.73)	7 (1.93)	
Other	21 (7.78)	15 (17.86)	59 (10.87)	42 (11.57)	
Donor					
Age, mean (SD), y	47.16 (14.81)	49.19 (14.39)	46.66 (17.13)	45.87 (16.00)	.37
BMI, mean (SD)	23.14 (3.39)	23.20 (3.21)	22.94 (3.62)	22.65 (3.16)	.26
Creatinine, mean (SD), mg/dL	0.93 (0.69-1.20)	0.96 (0.66-1.15)	0.89 (0.64-1.18)	0.87 (0.6-1.13)	.26
Ischemic time, mean (SD), h	20.84 (5.29)	20.10 (5.61)	20.06 (4.85)	20.21 (4.96)	.29
HLA mismatch, mean (SD)	3.10 (1.66)	2.85 (1.79)	3.03 (1.59)	3.01 (1.65)	.66
Immunosuppressants					
Tacrolimus	225 (83.33)	71 (84.52)	458 (84.35)	309 (85.1)	.63
Cyclosporin	42 (15.56)	12 (14.29)	83 (15.29)	53 (14.60)	
Rapamycin	3 (1.11)	1 (1.19)	2 (0.37)	1 (0.28)	
Tacrolimus level, ng/mL	6.29 (1.73)	6.36 (1.61)	6.47 (1.82)	6.39 (1.79)	.43
Laboratory values, mean (SD)					
White blood cells, 10^3^/μL	6.06 (2.25)	6.70 (2.69)	6.72 (3.11)	6.64 (2.74)	.02
Hemoglobin, g/dL	10.42 (1.86)	10.77 (1.88)	10.21 (2.02)	10.1 (2)	.02
Platelet, 10^3^/μL, median (IQR)	205.00 (167.00-254.00)	219.00 (187.25-261.75)	208.00 (168.00-271.00)	214.00 (174.00-264.00)	.69
Albumin, g/dL	4.25 (0.4)	4.16 (0.42)	4.19 (0.44)	4.18 (0.45)	.17
Globulin, g/dL	2.48 (0.41)	2.48 (0.41)	2.46 (0.43)	2.48 (0.42)	.95
ALT, U/L, median (IQR)	17.00 (12.00-23.00)	16.50 (11.00-28.75)	16.00 (11.00-25.00)	16.00 (10.00-25.00)	.95
AST, U/L, median (IQR)	14.00 (11.00-17.00)	15.00 (11.00-19.75)	14.00 (12.00-20.00)	15.00 (12.00-19.00)	.003
BUN, mg/dL	28.43 (11.88)	28.74 (13.14)	28.04 (13.84)	27.06 (14.93)	.57
Creatinine, mg/dL	1.44 (1.14-1.75)	1.43 (1.18-1.74)	1.30 (1.06-1.64)	1.25 (1-1.59)	<.001
UA, mg/dL, median (IQR)	5.65 (4.77-6.71)	5.78 (4.95-6.73)	5.63 (4.71-6.58)	5.56 (4.70-6.49)	.61
TG, mg/dL	2.19 (1.08)	1.52 (0.82)	2.00 (1.03)	1.29 (0.53)	<.001
HDL-C, mg/dL	1.15 (0.34)	1.42 (0.37)	1.31 (0.38)	1.51 (0.31)	.14
FBG, mg/dL	109.73 (31.17)	93.51 (9.91)	106.13 (38.38)	92.07 (14.05)	<.001
eGFR, mL/min/1.73 m^2^	59.78 (21.25)	59.52 (21.80)	62.58 (22.25)	66.85 (21.86)	.001
**Posttransplant follow-up**					
12 mo					
TG, mg/dL	2.08 (1.17)	1.38 (0.55)	1.81 (0.91)	1.27 (0.56)	<.001
HDL-C, mg/dL	1.17 (0.32)	1.49 (0.28)	1.36 (0.36)	1.64 (0.33)	.02
FBG, mg/dL	110.27 (32.07)	94.59 (10.99)	103.96 (31.89)	92.61 (12.79)	<.001
eGFR, mL/min/1.73 m^2^	62.41 (21.27)	61.78 (21.93)	64.13 (23.04)	69.28 (21.73)	<.001
18 mo					
TG, mg/dL	2.03 (1.24)	1.39 (0.64)	1.71 (0.82)	1.20 (0.44)	<.001
HDL-C, mg/dL	1.17 (0.32)	1.49 (0.28)	1.36 (0.36)	1.64 (0.33)	.02
FBG, mg/dL	108.47 (33.15)	93.33 (11.53)	102.52 (25.59)	91.71 (10.45)	<.001
eGFR, mL/min/1.73 m^2^	63.58 (22.64)	63.29 (21.76)	65.76 (22.80)	69.36 (21.99)	.02
24 mo					
TG, mg/dL	2.17 (1.43)	1.49 (0.61)	1.73 (0.94)	1.25 (0.63)	<.001
HDL-C, mg/dL	1.18 (0.30)	1.45 (0.20)	1.46 (0.38)	1.50 (0.45)	.35
FBG, mg/dL	114.41 (37.48)	93.15 (10.63)	100.72 (21.62)	92.79 (10.81)	<.001
eGFR, mL/min/1.73 m^2^	63.63 (23.42)	64.20 (21.38)	66.75 (22.98)	70.26 (21.33)	.02

Abbreviations: ADPKD, autosomal dominant polycystic kidney disease; ALT, alanine aminotransferase; AST, aspartate aminotransferase; BMI, body mass index (calculated as weight in kilograms divided by height in meters squared); BUN, blood urea nitrogen; DKD, diabetic kidney disease; eGFR, estimated glomerular filtration rate; FBG, fasting blood glucose; HDL-C, high-density lipoprotein cholesterol; HLA, human leucocyte antigen; MHNO, metabolically healthy not overweight or obese; MHO, metabolically healthy overweight or obese; MUNO, metabolically unhealthy not overweight or obese; MUO, metabolically unhealthy overweight or obese; PRA, panel reactive antibody; TC, total cholesterol; TG, triglyceride; UA, uric acid.

SI conversion factors: To convert albumin to grams per liter, multiply by 10; ALT and AST to microkatals per liter, multiply by 0.0167; BUN to millimoles per liter, multiply by 0.357; creatinine to micromoles per liter, multiply by 88.4; FBG to millimoles per liter, multiply by 0.0555; hemoglobin and white blood cells to 10^9^/L, multiply by 1; TG to millimoles per liter, multiply by 0.0113; TC and HDL-C to millimoles per liter, multiply by 0.0259; UA to millimoles per liter, multiply by 0.0595.

**Table 3.** Risk for GFD Based on Metabolic Phenotype Subgroups

Subgroup	GFD incidence , No./total No. (%)	Crude OR (95% CI)	*P* value	Model 1 OR (95% CI)^a^	*P* value	Model 2 OR (95% CI)^b^	*P* value	Model 3 OR (95% CI)^c^	*P* value
MHNO	22/363 (6.06)	1 [Reference]	NA	1 [Reference]	NA	1 [Reference]	NA	1 [Reference]	NA
MUNO	58/543 (10.68)	1.85 (1.11-3.09)	.02	1.78 (1.06-2.98)	.03	1.92 (1.11-3.32)	.02	1.91 (1.11-3.31)	.02
MHO	9/84 (10.71)	1.86 (0.82-4.20)	.14	1.86 (0.81-4.26)	.14	2.39 (1.02-5.62)	.045	2.37 (1.01-5.57)	.048
MUO	38/270 (14.07)	2.54 (1.46-4.40)	.001	2.47 (1.40-4.36)	.002	2.76 (1.50-5.05)	.001	2.76 (1.50-5.06)	.001

Abbreviations: GFD, graft function deterioration; MHNO, metabolically healthy not overweight or obese; MHO, metabolically healthy overweight or obese; MUNO, metabolically unhealthy not overweight or obese; MUO, metabolically unhealthy overweight or obese; NA, not applicable; OR, odds ratio.

^a^ Adjusted for age, sex, and estimated glomerular filtration rate at baseline.

^b^ Adjusted for model 1 plus panel reactive antibody, hemoglobin, albumin, and primary disease.

^c^ Adjusted for model 2 plus donor age, donor creatinine, donor body mass index, and human leucocyte antigen mismatch.

### Risk for GFD by Specific Metabolic Components

After adjusting for confounding factors in multivariable analysis, all metabolic components, except for elevated triglyceride, showed an independent association with an increased risk of GFD. When treated as continuous variables, metabolic conditions (OR per additional condition, 1.40; 95% CI, 1.20-1.63; *P* < .001) and BMI (OR per 1-unit increase, 1.90; 95% CI, 1.03-1.15; *P* = .002) were associated with an increased risk of GFD (Table 4). We used restricted cubic splines to visualize the relation of BMI with the primary outcome. A multivariable adjusted logistic regression model showed a *J*-shaped association between BMI and GFD (*P* for nonlinearity = .001) (Figure).

**Table 4.** Risk for GFD Based on the Prevalence of Specific Metabolic Components

Metabolic condition	GFD incidence, No./total No. (%)	Crude OR (95% CI)	*P* value	Model 1 OR (95% CI)^a^	*P* value	Model 2 OR (95% CI)^b^	*P* value	Model 3 OR (95% CI)^c^	*P* value
Hyperglycemia	50/325 (15.38)	1.91 (1.30-2.81)	.001	2.08 (1.36-3.17)	.001	2.04 (1.34-3.12)	.001	2.01 (1.31-3.07)	.001
Hypertension	109/978 (11.15)	1.79 (1.06-3.03)	.03	1.87 (1.10-3.16)	.02	1.95 (1.15-3.31)	.01	1.93 (1.13-3.28)	.015
Elevated TG	79/736 (10.73)	0.89 (0.59-1.34)	.57	0.87 (0.57-1.31)	.50	0.91 (0.60-1.39)	.67	0.92 (0.61-1.40)	.70
Low HDL-C	57/410 (13.90)	1.79 (1.19-2.69)	.005	1.74 (1.15-2.61)	.008	1.71 (1.13-2.56)	.01	1.67 (1.11-2.52)	.01
BMI	NA^d^	1.08 (1.03-1.14)	.002	1.08 (1.02-1.14)	.004	1.09 (1.03-1.15)	.002	1.09 (1.03-1.15)	.002
No. of Metabolic conditions	NA^d^	1.39 (1.20-1.61)	<.001	1.39 (1.19-1.62)	<.001	1.41 (1.21-1.65)	<.001	1.40 (1.20-1.63)	<.001

Abbreviations: BMI, body mass index (calculated as weight in kilograms divided by height in meters squared); GFD, graft function deterioration; HDL-C, high-density lipoprotein cholesterol; NA, not applicable; OR, odds ratio; TG, triglyceride.

^a^ Adjusted for age, sex, and estimated glomerular filtration rate at baseline.

^b^ Adjusted for model 1 plus panel reactive antibody, hemoglobin, albumin, and primary disease.

^c^ Adjusted for model 2 plus donor age, donor creatinine, donor body mass index, and human leucocyte antigen mismatch.

^d^ BMI and number of metabolic conditions were treated as continuous variable.

**Figure.**
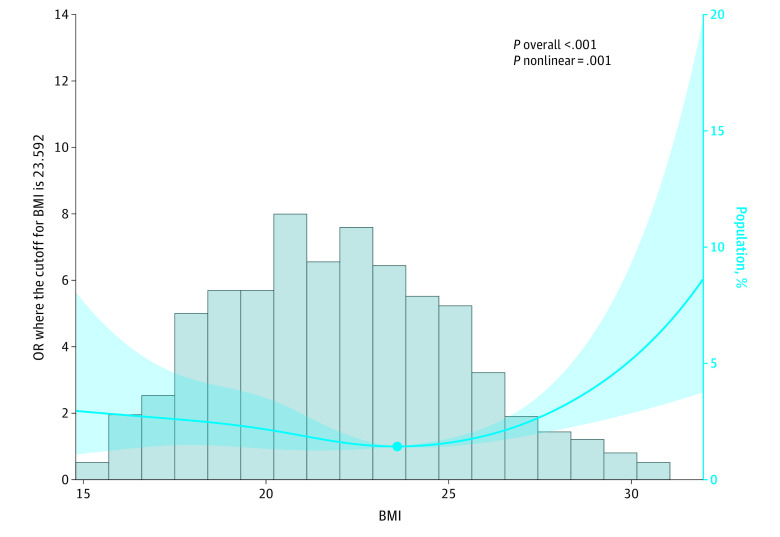
Restricted Cubic Spline Curve for the Association of Body Mass Index (BMI) With Risk of Graft Function Deterioration The logistic regression was adjusted for sex, age, primary disease, and other metabolic components. The model had 4 knots, located at the 5th, 35th, 65th, and 95th percentiles. Bars represent the distribution of BMI (calculated as weight in kilograms divided by height in meters squared); lines, estimated odds ratios (ORs) of BMI for the risk of graft function deterioration; and shading, 95% CIs. The risk of graft function progression initially decreased as BMI increased until reaching 23.592 (reference). However, beyond this point, the risk started to increase.

### Sensitivity Analysis

Sensitivity analysis was conducted excluding individuals with diabetes at transplant and those with hypertensive nephrosclerosis or diabetic kidney disease as the primary disease. The results remained consistent, showing that the MUNO, MHO, and MUO subgroups had an increased risk for the primary outcome compared with the MHNO subgroup (eTable 4 and eTable 5 in Supplement 1). Further analysis using a 20% decline in eGFR to define GFD yielded consistent results. However, when we used a cutoff of 30% decline in eGFR, the result of the MHO group was attenuated (eTable 6 and eTable 7 in Supplement 1).

## Discussion

In this cohort study, we investigated the associations of overweight or obesity, metabolic conditions, and GFD in recipients of kidney transplant recipients from multiple centers in China. We found that overweight or obesity and metabolic conditions were significantly associated with adverse graft outcomes. Among the different metabolic phenotypes studied, MUO was associated with the highest risk of GFD. Interestingly, the MHO phenotype also was associated with a higher risk compared with the MHNO phenotype, although the 95% CI was wide and some uncertainty remains. Moreover, the number of metabolic conditions was associated with an increased risk of GFD. Regarding the specific metabolic elements, overweight or obesity, hypertension, hyperglycemia, and decreased HDL-C level were all independently associated with GFD, but elevated triglyceride was not. A nonlinear association was observed between BMI and risk of GFD.

Previous studies have investigated the association of overweight and obesity with graft outcomes in recipients of kidney transplant. The association of BMI with medium- and long-term outcomes has been controversial.^28,30,31,32,33,34,35^ For instance, a study by Hoogeveen et al^30^ found that recipients of transplant with BMI exceeding 30 had 20% to 40% increased risk of mortality and graft loss compared with recipients of transplant with BMI within reference range. A meta-analysis by Sood et al^34^ found a higher incidence of delayed graft function, acute rejection, graft failure, and mortality among recipients of kidney transplant with obesity. On the other hand, Tsapepas et al^28^ conducted a study of recipients of kidney transplant covering a broad spectrum of BMI categories, and found that high BMI was not associated with adverse long-term graft survival. Another systematic review and meta-analysis^35^ found that obesity was associated with an increased risk of delayed graft function but not acute rejection. Additionally, associations of obesity with increased graft loss and mortality were only observed in studies involving patients who underwent transplantation prior to the year 2000. The finding of these studies should be further verified with more high quality studies. CAD is one of the primary causes for graft failure after 12 months in individuals who have undergone kidney transplantation. Regarding potential contributors to CAD, both immune-related and non–immune-related risk factors have been suggested. Elements of metabolic disorders, which are significant non–immune-related factors, have been found to be associated with the onset of CAD.^36^ The graft histologic lesions observed in CAD are similar to those seen in native kidneys affected by overweight and obesity and metabolic disturbances, including interstitial fibrosis, tubular atrophy, and arterial sclerosis. A study by de Vries et al^25^ found an association between metabolic conditions and suboptimal graft function, with systolic blood pressure and triglyceride levels being specifically associated with adverse renal outcomes.^25^ A study by Porrini et al^26^ involving 230 recipients of kidney transplant found that individuals diagnosed with a metabolic disorder exhibited a higher incidence of CAD. When examining the different elements of metabolic disorders, diastolic blood pressure and BMI showed significant associations with CAD. These studies^25,26^ found variations in the specific metabolic elements that were associated with CAD, possibly due to unmeasured factors and differences in study populations, as the study by Porrini et al^26^ was restricted to patients without diabetes.

In this study, we found consistent results with some of previous investigations: being overweight or obese or having metabolic disorder was associated with poor graft outcomes. Notably, the highest risk for GFD was observed in patients having both overweight or obesity and metabolic disorder; these patients had increased numbers of unfavorable risk factors, which can hasten GFD. Our finding of an association between the increasing number of metabolic conditions and an elevation in the risk of GFD is also consistent with previous literature. Furthermore, our results support the notion that not all metabolic components contribute equally to CAD, although the specific contributing components differed from previous investigations. Our study had the advantage of a relatively large sample size, allowing us to adjust for important confounding factors in multivariable analyses. Additionally, our study assessed metabolic conditions during the earlier period (from 1 month through 6 months) after transplantation, which differs from previous studies^25,26^ that evaluated these conditions at the baseline of 1 year after transplantation. It is worth noting that metabolic status can be temporarily impacted by short-term factors, such as acute rejection, infection, delayed graft function, and the adjustment of immunosuppressant dosages, particularly in the first year following transplantation. To account for fluctuating metabolic status during the early posttransplantation phase, we took the mean values of metabolic component levels into consideration. This approach helps to capture the overall metabolic burden experienced during this period. Moreover, we observed a nonlinear association between BMI and adverse graft outcome. The BMI cutoff of 24 used for the Chinese population in this study was lower than what may be considered the BMI reference range in some other racial and ethnic groups. Additional studies are needed to verify and further explore the research question.

Previous studies have suggested that individuals who are obese but do not have metabolic irregularities may still have adipose tissue functioning within reference ranges, typical patterns of adipokine secretion, and stable insulin sensitivity.^37^ However, recent studies have questioned whether individuals with this metabolic phenotype are truly healthy. A study conducted by Xu et al^38^ found that individuals with MHO may have a higher likelihood of developing glucose anomalies and high blood pressure in the future. Zhao et al^39^ observed that patients with the MHO phenotype exhibited signs of subclinical cardiac dysfunction. Another study by Yun et al^40^ found that individuals with MHO also had a heightened risk for CKD progression. These results indicate that individuals with this unique metabolic profile might still be susceptible to potential adverse outcomes. Taking these factors into account, we examined different overweight and obesity subtypes to determine whether outcomes might differ based on metabolic condition. Our study expands the evidences to include the kidney transplant population, revealing that the individuals with MHO also had an increased risk of adverse graft outcome compared with MHNO. It has been proposed that the MHO phenotype may represent a transitional phase toward a higher metabolic risk in the future. Overweight and obesity may act as a triggering factor for metabolic disorder, which would further exacerbate the risk of CAD progression. Further observation of cardiometabolic factors over time will help elucidate the mechanistic link and explain the adverse outcomes in individuals with MHO.

### Limitations

Our study has several limitations. First, the prevalence of different metabolic phenotypes can vary according to the specific criteria used to define them. In this study, MHO was characterized by overweight or obesity with less than 2 components of metabolic disorder, which aligns with previous studies.^41^ Second, accurate data regarding the underlying cause of GFD were unavailable due to the limited number of patients who underwent graft pathologic examination. Third, our research was conducted exclusively among individuals from China, and we categorized individuals as overweight or obese using the domestic threshold, which is lower than the general population criteria set by the World Health Organization (25.0).^42^ Previous studies have suggested that Asian populations may have a greater risk of adverse outcomes associated with BMI, even when under a lower BMI range. Hence, our findings may have limited applicability to other ethnic groups. Additionally, due to a limited number of deaths during the follow-up, we used kidney allograft function as a surrogate end point. It is crucial to conduct long-term observations using a large sample size to assess the potential associations with more definite outcomes.

## Conclusions

In this cohort study of adult recipients of cadaveric kidney transplant, overweight and obesity and metabolic disorder were associated with GFD in recipients of kidney transplant. Furthermore, our findings suggest that MHO should not be overlooked as a risk profile, since it was associated with an increased risk of GFD, although the 95% CI was wide and some uncertainty remains. Our study provides a justification for regular monitoring of metabolic risk profiles early after kidney transplantation. It is essential to conduct additional studies with larger sample sizes and longer follow-up periods to validate our findings.

## Notes

Supplement 1.
eMethods.
**eTable 1.** Association of Weight and Metabolic Status With the Risk of Graft Function Deterioration **eTable 2.** Risk for the Composite Outcome of Graft Function Deterioration or Graft Loss Among the Metabolic Phenotypes **eTable 3.** Generalized Estimating Equation Analysis of Metabolic Phenotypes With Graft Function Deterioration **eTable 4.** Risk for Graft Function Deterioration Among the Metabolic Phenotypes Excluding Individuals With Diabetes at Transplant **eTable 5.** Risk for Graft Function Deterioration Among the Metabolic Phenotypes Excluding Individuals With Hypertensive Nephrosclerosis or Diabetic Kidney Disease as Primary Disease **eTable 6.** Risk for Graft Function Deterioration of ≥20% Among the Metabolic Phenotypes **eTable 7.** Risk for Graft Function Deterioration of ≥30% Among the Metabolic Phenotypes **eFigure 1.** Assessment for Eligibility **eFigure 2.** Change in eGFR Between Six Months and Two Years Post-transplant Among the Metabolic Phenotypes **eFigure 3.** Kaplan-Meier Curve for eGFR Decline Among the Metabolic Phenotypes **eFigure 4.** Kaplan-Meier Curve for Overall Graft Survival Among the Metabolic Phenotypes
eReferences.

Supplement 2.
Data Sharing Statement
