# Supplementary material for: Metabolic Risk Profile and Graft Function Deterioration 2 Years After Kidney Transplant
Source: JAMA Netw Open. 2023 Dec 27;6(12):e2349538. doi: 10.1001/jamanetworkopen.2023.49538 (PMC10753396; doi:10.1001/jamanetworkopen.2023.49538)
Supplement: Supplement 1. — eMethods. eTable 1. Association of Weight and Metabolic Status With the Risk of Graft Function Deterioration eTable 2. Risk for the Composite Outcome of Graft Function Deterioration or Graft Loss Among the Metabolic Phenotypes eTable 3. Generalized Estimating Equation Analysis of Metabolic Phenotypes With Graft Function Deterioration eTable 4. Risk for Graft Function Deterioration Among the Metabolic Phenotypes Excluding Individuals With Diabetes at Transplant eTable 5. Risk for Graft Function Deterioration Among the Metabolic Phenotypes Excluding Individuals With Hypertensive Nephrosclerosis or Diabetic Kidney Disease as Primary Disease eTable 6. Risk for Graft Function Deterioration of ≥20% Among the Metabolic Phenotypes eTable 7. Risk for Graft Function Deterioration of ≥30% Among the Metabolic Phenotypes eFigure 1. Assessment for Eligibility eFigure 2. Change in eGFR Between Six Months and Two Years Post-transplant Among the Metabolic Phenotypes eFigure 3. Kaplan-Meier Curve for eGFR Decline Among the Metabolic Phenotypes eFigure 4. Kaplan-Meier Curve for Overall Graft Survival Among the Metabolic Phenotypes eReferences. [file jamanetwopen-e2349538-s001.pdf]

Supplemental Online Content

Yan J, Yang X, Wang J, et al. Metabolic risk profile and graft function deterioration 2 years after kidney transplant. *JAMA Netw Open*. 2023;6(12):e2349538. doi:10.1001/jamanetworkopen.2023.49538

eMethods.
eTable 1. Association of Weight and Metabolic Status With the Risk of Graft Function Deterioration
eTable 2. Risk for the Composite Outcome of Graft Function Deterioration or Graft Loss Among the Metabolic Phenotypes
eTable 3. Generalized Estimating Equation Analysis of Metabolic Phenotypes With Graft Function Deterioration
eTable 4. Risk for Graft Function Deterioration Among the Metabolic Phenotypes Excluding Individuals With Diabetes at Transplant
eTable 5. Risk for Graft Function Deterioration Among the Metabolic Phenotypes Excluding Individuals With Hypertensive Nephrosclerosis or Diabetic Kidney Disease as Primary Disease
eTable 6. Risk for Graft Function Deterioration of ⩾20% Among the Metabolic Phenotypes
eTable 7. Risk for Graft Function Deterioration of ⩾30% Among the Metabolic Phenotypes
eFigure 1. Assessment for Eligibility
eFigure 2. Change in eGFR Between 6 Months and 2 Years Posttransplant Among the Metabolic Phenotypes
eFigure 3. Kaplan-Meier Curve for eGFR Decline Among the Metabolic Phenotypes
eFigure 4. Kaplan-Meier Curve for Overall Graft Survival Among the Metabolic Phenotypes
eReferences.

This supplemental material has been provided by the authors to give readers additional information about their work.

eMethods

Study participants

The “Construction of solid organ transplantation database and biobank” project was a prospective cohort study incorporating multiple tertiary-care hospitals in China. The summary of the study protocol is presented elsewhere (Chinese Clinical Trial registration number, ChiCTR2100052682). Briefly, the project utilized unified standards to construct a large organ transplantation data management platform integrating multi-level data such as clinical phenotype, disease diagnosis, treatment data, and follow-up information.

Definitions of weight and metabolic status

Overweight/obesity in this context refers to individuals with a body mass index (BMI) equal to or greater than 24.0 kg/m^2, as per the weight criteria outlined by the Working Group on Obesity in China [1]. The threshold was also established following the research conducted by the Cooperative Meta-analysis Group of China Obesity Task Force [2]. The definitive criteria for defining metabolic disorder are not yet established. However, various studies have utilized specific indicators such as hypertension; dyslipidemia, including elevated triglyceride (TG) or low-density lipoprotein cholesterol, or decreased high-density lipoprotein cholesterol (HDL-C) levels; changes in glucose metabolism, evidenced by increased fasting plasma glucose, hemoglobin A1c levels, and a history of diabetes mellitus. In this study, we focused on four clinical and laboratory parameters based on the ATP-III definition [3], including hypertension, low HDL-C, elevated TG levels, and hyperglycemia.

It is important to note that during the early post-transplantation phase, metabolic status may be temporarily impacted by factors such as acute rejection, opportunistic infection, delayed graft function (DGF), and the administration of higher doses of calcineurin inhibitors and corticosteroids. In order to prevent the bias in determining abnormal metabolic status based on a single evaluation at a specific timepoint, we established the metabolic burden by taking into account the average value of metabolic parameters from one month to six months following the transplantation. Specifically, the criteria were as follows: (1) hypertension defined as average systolic blood pressure ⩾ 130 mmHg, or average diastolic blood pressure ⩾ 85 mmHg, or use of antihypertensive medications; (2) average fasting plasma glucose level ⩾ 5.6 mmol/L, or history of type 2 diabetes, or use of hypoglycemics; (3) average TG level ⩾ 1.7 mmol/L or use of lipid-lowering drugs; (4) average HDL-C level < 1.04 mmol/L in men (< 1.3 mmol/L in women). The waist circumference criterion was not used to classify MHO because of its collinearity with body mass index [4]. Metabolic disorder was defined as the presence of two or more of these parameters. Similarly, overweight/obesity was defined as average BMI ⩾ 24.0 kg/m^2. As the protocols in our kidney transplant centers, outpatient follow-up involves regular visits and blood tests, with the frequency gradually decreasing over time. During the initial one to three months after transplantation, patients usually have weekly or biweekly visits. This frequency then transitions to monthly visits until six months. Such a follow-up schedule ensures sufficient assessment of metabolic parameters from one month to six months post-transplantation. Patients were categorized into four phenotypes according to the baseline assessment: (1) metabolically healthy non-overweight/obesity (MHNO), (2) metabolically healthy overweight/obesity (MHO), (3) metabolically unhealthy non-overweight/obesity (MUNO), or (4) metabolically unhealthy overweight/obesity (MUO).

© 2023 Yan J et al. *JAMA Network Open.*

Outcomes

In order to observe long-term outcomes after transplantation, it is necessary to either have a large study population or extend the follow-up period, as the rates of graft failure or mortality within the initial three years following transplantation are typically low. As graft function serves as a well-established surrogate estimate for predicting the long-term survival of renal allografts, we utilized a modified approach that assessed changes in graft function over a specific time frame as the surrogate endpoint. Based on the report of Clayton et al. [5], we defined the primary outcome as an equal to or greater than 25% decline in estimated glomerular filtration rate (eGFR) between six months and two years post-transplant. For patients who had graft loss before two years, the last eGFR level was used for analysis. Considering the fact that after kidney transplantation the course of kidney function is more subject to acute transient recipient events, such as infection, rejection, and changes in immunosuppressive therapy. Acute kidney injury at early post-transplant stage is not rare, especially within one year post transplant, and can be affected by numerous factors [6]. Thus, we selected logistic regression analysis as the primary method to evaluate eGFR decline between six months and two years post-transplant, while using Generalized Estimating Equations (GEE) and time-to-event analysis as additional approaches. In line with the protocols followed in our kidney transplant centers, the evaluation of metabolic parameters and eGFR is usually conducted at three-month to two-month intervals during the six-month to one-year period after transplant. Subsequently, the frequency is gradually reduced to once every six months, based on the individual's health condition. We employed the CKD Epidemiology Collaboration (CKD-EPI) equation to calculate eGFR [7].

Immunosuppression

All recipients received a combination of either rabbit-antihuman-thymocyte immunoglobulin or basiliximab, along with methylprednisolone, as an initial immunosuppression therapy. This was followed by the administration of oral corticosteroids, mycophenolate mofetil, and typically calcineurin inhibitors as maintenance immunosuppressants. Prednisone was started with 20 mg/d, then rapidly tapered to 5-10 mg/d. The target for tacrolimus trough concentration level was 6-10 ng/ml during the first 3 months postoperatively, then tapered to 4-8 ng/ml by 12 months postoperatively. The cyclosporine trough concentration was maintained at 150-250 ng/ml during the first 3 months postoperatively, then tapered to 100-150 ng/ml by 12 months postoperatively. The concentration of mycophenolic acid was controlled to remain within the range of 30-60 mg·h/l, as indicated by the area under the curve.

© 2023 Yan J et al. *JAMA Network Open.*

eTable 1. Association of weight and metabolic status with the risk of graft function deterioration

	Incidence of endpoint No./total No. (%)	Crude	P value	Model 1	P value	Model 2	P value	Model 3	P value
**Non-Overweight/Obesity**	80/906(8.83)	Ref		Ref		Ref		Ref	
**Overweight/Obesity**	47/354(13.28)	1.58(1.08-2.32)	0.019	1.56(1.05-2.32)	0.027	1.64(1.10-2.44)	0.015	1.64(1.10-2.44)	0.016
**Without Metabolic disorder**	32/449(7.13)	Ref		Ref		Ref		Ref	
**Metabolic disorder**	95/811(11.43)	1.73 (1.14-2.63)	0.010	1.68 (1.10-2.57)	0.017	1.74 (1.13-2.66)	0.012	1.71(1.12-2.63)	0.014

Model 1: Adjusted for age and sex

Model 2: Adjusted for Model 1 plus PRA, creatinine, hemoglobin, albumin, and primary disease

Model 3: Adjusted for Model 2 plus donor age, creatinine, and HLA mismatch

© 2023 Yan J et al. *JAMA Network Open.*

eTable 2. Risk for the composite outcome of graft function deterioration or graft loss among the metabolic phenotypes

	Incidence of endpoint No./total No. (%)	Crude	P value	Model 1	P value	Model 2	P value	Model 3	P value
**MHNO**	28/363(7.71)	Ref		Ref		Ref		Ref	
**MUNO**	67/543(12.34)	1.68(1.06-2.68)	0.027	1.66(1.02-2.69)	0.040	1.73(1.04-2.88)	0.034	1.74(1.05-2.90)	0.033
**MHO**	11/84(13.10)	1.80(0.86-3.79)	0.120	1.78(0.81-3.90)	0.152	2.14(0.96-4.79)	0.064	2.14(0.96-4.80)	0.064
**MUO**	39/270(14.44)	2.02(1.21-3.38)	0.007	2.14(1.24-3.67)	0.006	2.38(1.34-4.21)	0.003	2.39(1.35-4.26)	0.003

MUO: metabolically unhealthy overweight/obesity; MHO: metabolically healthy overweight/obesity; MUNO: metabolically unhealthy non-overweight/obesity; MHNO: metabolically healthy non-overweight/obesity

Model 1: Adjusted for age, sex, and eGFR at baseline

Model 2: Adjusted for Model 1 plus PRA, hemoglobin, albumin, and primary disease

Model 3: Adjusted for Model 2 plus donor age, donor creatinine, donor BMI, and HLA mismatch

© 2023 Yan J et al. *JAMA Network Open.*

eTable 3. Generalized estimating equation analysis of metabolic phenotypes with graft function deterioration

	Crude	P value	Model 1	P value	Model 2	P value	Model 3	P value
**MHNO**	Ref		Ref		Ref		Ref	
**MUNO**	1.50(1.06-2.12)	0.023	1.45(1.03-2.05)	0.036	1.49(1.05-2.11)	0.026	1.49(1.05-2.11)	0.025
**MHO**	1.66(0.95-2.91)	0.076	1.67(0.95-2.93)	0.078	1.77(1.08-2.89)	0.045	1.78(1.09-2.90)	0.043
**MUO**	1.88(1.27-2.79)	0.002	1.84(1.24-2.74)	0.003	1.95(1.30-2.92)	0.001	1.95(1.30-2.93)	0.010

MUO: metabolically unhealthy overweight/obesity; MHO: metabolically healthy overweight/obesity; MUNO: metabolically unhealthy non-overweight/obesity; MHNO: metabolically healthy non-overweight/obesity

Model 1: Adjusted for age, sex, and eGFR at baseline

Model 2: Adjusted for Model 1 plus PRA, hemoglobin, albumin, and primary disease

Model 3: Adjusted for Model 2 plus donor age, donor creatinine, donor BMI, and HLA mismatch

© 2023 Yan J et al. *JAMA Network Open.*

eTable 4. Risk for graft function deterioration among the metabolic phenotypes excluding individuals with diabetes at transplant

	Incidence of endpoint No./total No. (%)	Crude	P value	Model 1	P value	Model 2	P value	Model 3	P value
**MHNO**	19/350(5.43)	Ref		Ref		Ref		Ref	
**MUNO**	33/363(9.09)	1.74(0.97-3.13)	0.063	1.67(0.93-3.01)	0.089	1.74(0.93-3.25)	0.084	1.76(0.94-3.29)	0.080
**MHO**	8/81(9.88)	1.91(0.81-4.53)	0.142	2.00(0.83-4.82)	0.124	2.59(1.04-6.44)	0.041	2.57(1.03-6.42)	0.043
**MUO**	16/140(11.43)	2.25(1.12-4.51)	0.023	2.26(1.11-4.58)	0.024	2.36(1.10-5.06)	0.028	2.36(1.09-5.09)	0.029

MUO: metabolically unhealthy overweight/obesity; MHO: metabolically healthy overweight/obesity; MUNO: metabolically unhealthy non-overweight/obesity; MHNO: metabolically healthy non-overweight/obesity;

Model 1: Adjusted for age, sex, and eGFR at baseline

Model 2: Adjusted for Model 1 plus PRA, hemoglobin, albumin, and primary disease

Model 3: Adjusted for Model 2 plus donor age, donor creatinine, donor BMI, and HLA mismatch

© 2023 Yan J et al. *JAMA Network Open.*

eTable 5. Risk for graft function deterioration among the metabolic phenotypes excluding individuals with hypertensive nephrosclerosis or diabetic kidney disease as primary disease

	Incidence of endpoint No./total No. (%)	Crude	P value	Model 1	P value	Model 2	P value	Model 3	P value
**MHNO**	22/356(6.18)	Ref		Ref		Ref		Ref	
**MUNO**	51/501(10.18)	1.73(1.03-2.91)	0.038	1.70(1.00-2.87)	0.048	1.78(1.05-3.03)	0.032	1.80(1.06-3.07)	0.030
**MHO**	8/77(10.39)	2.02(0.89-4.58)	0.092	2.00(0.87-4.60)	0.102	2.30(0.99-5.34)	0.052	2.35(1.01-5.45)	0.048
**MUO**	32/238(13.44)	2.37(1.34-4.20)	0.003	2.34(1.30-4.22)	0.004	2.61(1.44-4.74)	0.002	2.61(1.44-4.75)	0.002

MUO: metabolically unhealthy overweight/obesity; MHO: metabolically healthy overweight/obesity; MUNO: metabolically unhealthy non-overweight/obesity; MHNO: metabolically healthy non-overweight/obesity;

Model 1: Adjusted for age, sex, and eGFR at baseline

Model 2: Adjusted for Model 1 plus PRA, hemoglobin, albumin, and primary disease

Model 3: Adjusted for Model 2 plus donor age, donor creatinine, donor BMI, and HLA mismatch

© 2023 Yan J et al. *JAMA Network Open.*

eTable 6. Risk for graft function deterioration of ⩾20% among the metabolic phenotypes

	Incidence of endpoint No./total No. (%)	Crude	P value	Model 1	P value	Model 2	P value	Model 3	P value
**MHNO**	32/363(8.82)	Ref		Ref		Ref		Ref	
**MUNO**	74/543(13.63)	1.63(1.05-2.53)	0.028	1.64(1.05-2.55)	0.030	1.75(1.10-2.79)	0.018	1.75(1.10-2.79)	0.019
**MHO**	12/84(14.29)	1.72(0.85-3.51)	0.133	1.84(0.89-3.79)	0.098	2.17(1.04-4.55)	0.040	2.18(1.04-4.58)	0.039
**MUO**	43/270(15.93)	1.96(1.20-3.19)	0.007	2.05(1.24-3.39)	0.005	2.21(1.30-3.77)	0.003	2.33(1.30-3.79)	0.003

MUO: metabolically unhealthy overweight/obesity; MHO: metabolically healthy overweight/obesity; MUNO: metabolically unhealthy non-overweight/obesity; MHNO: metabolically healthy non-overweight/obesity

Model 1: Adjusted for age, sex, and eGFR at baseline

Model 2: Adjusted for Model 1 plus PRA, hemoglobin, albumin, and primary disease

Model 3: Adjusted for Model 2 plus donor age, donor creatinine, donor BMI, and HLA mismatch

© 2023 Yan J et al. *JAMA Network Open.*

eTable 7. Risk for graft function deterioration of ⩾30% among the metabolic phenotypes

	Incidence of endpoint No./total No. (%)	Crude	P value	Model 1	P value	Model 2	P value	Model 3	P value
**MHNO**	15/363(4.13)	Ref		Ref		Ref		Ref	
**MUNO**	42/543(7.73)	1.95(1.06-3.56)	0.031	1.85(1.00-3.41)	0.049	1.92(1.01-3.63)	0.046	1.93(1.02-3.66)	0.044
**MHO**	7/84(8.33)	2.11(0.83-5.35)	0.116	2.03(0.79-5.21)	0.143	2.54(0.96-6.70)	0.059	2.59(0.98-6.83)	0.055
**MUO**	24/270(8.89)	2.26(1.16-4.40)	0.016	2.09(1.06-4.14)	0.035	2.31(1.13-4.74)	0.022	2.27(1.10-4.68)	0.026

MUO: metabolically unhealthy overweight/obesity; MHO: metabolically healthy overweight/obesity; MUNO: metabolically unhealthy non-overweight/obesity; MHNO: metabolically healthy non-overweight/obesity

Model 1: Adjusted for age, sex, and eGFR at baseline

Model 2: Adjusted for Model 1 plus PRA, hemoglobin, albumin, and primary disease

Model 3: Adjusted for Model 2 plus donor age, donor creatinine, donor BMI, and HLA mismatch

© 2023 Yan J et al. *JAMA Network Open.*

eFigure 1. Assessment for eligibility

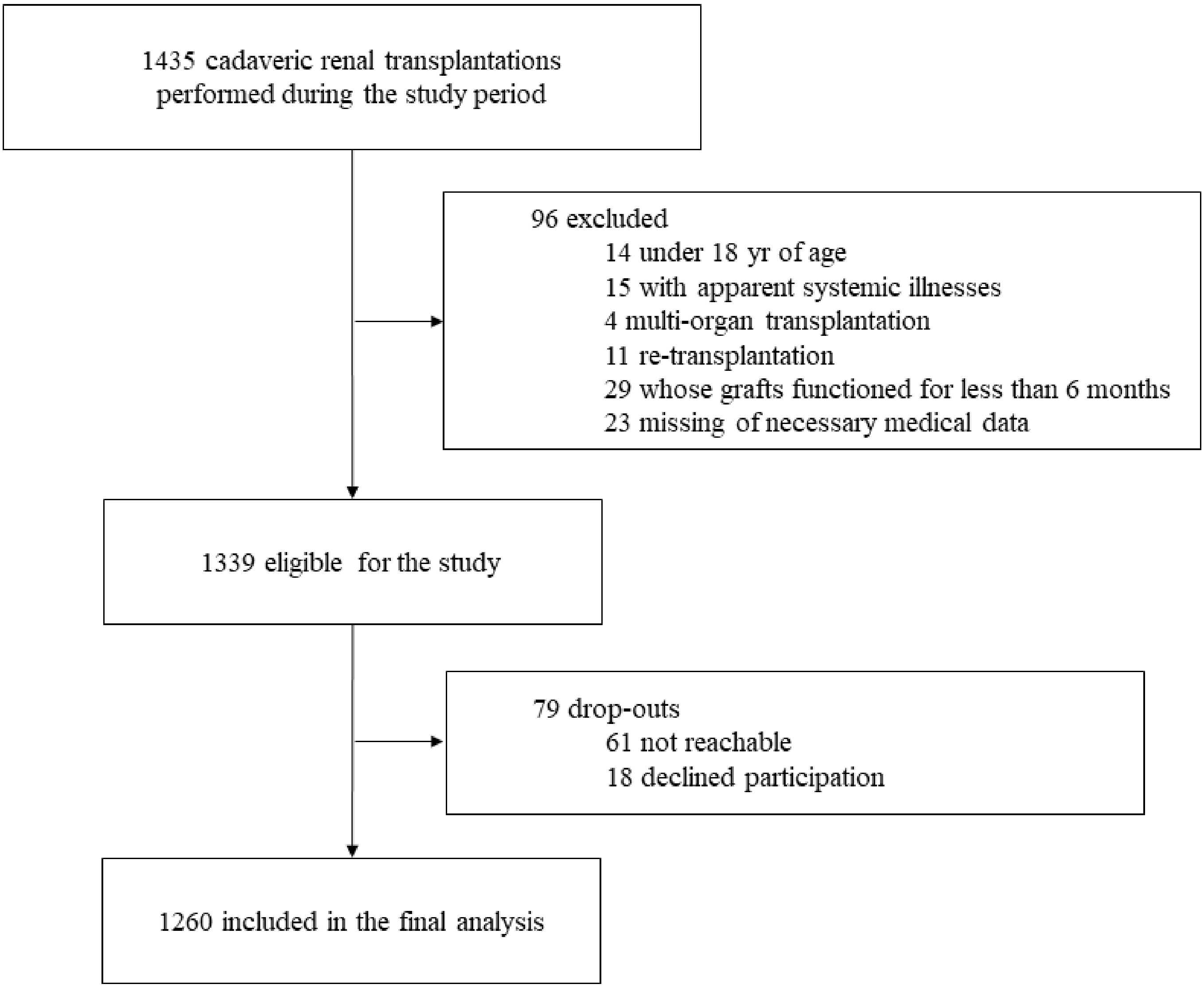

© 2023 Yan J et al. *JAMA Network Open.*

eFigure 2. Change in eGFR between six months and two years post-transplant among the metabolic phenotypes

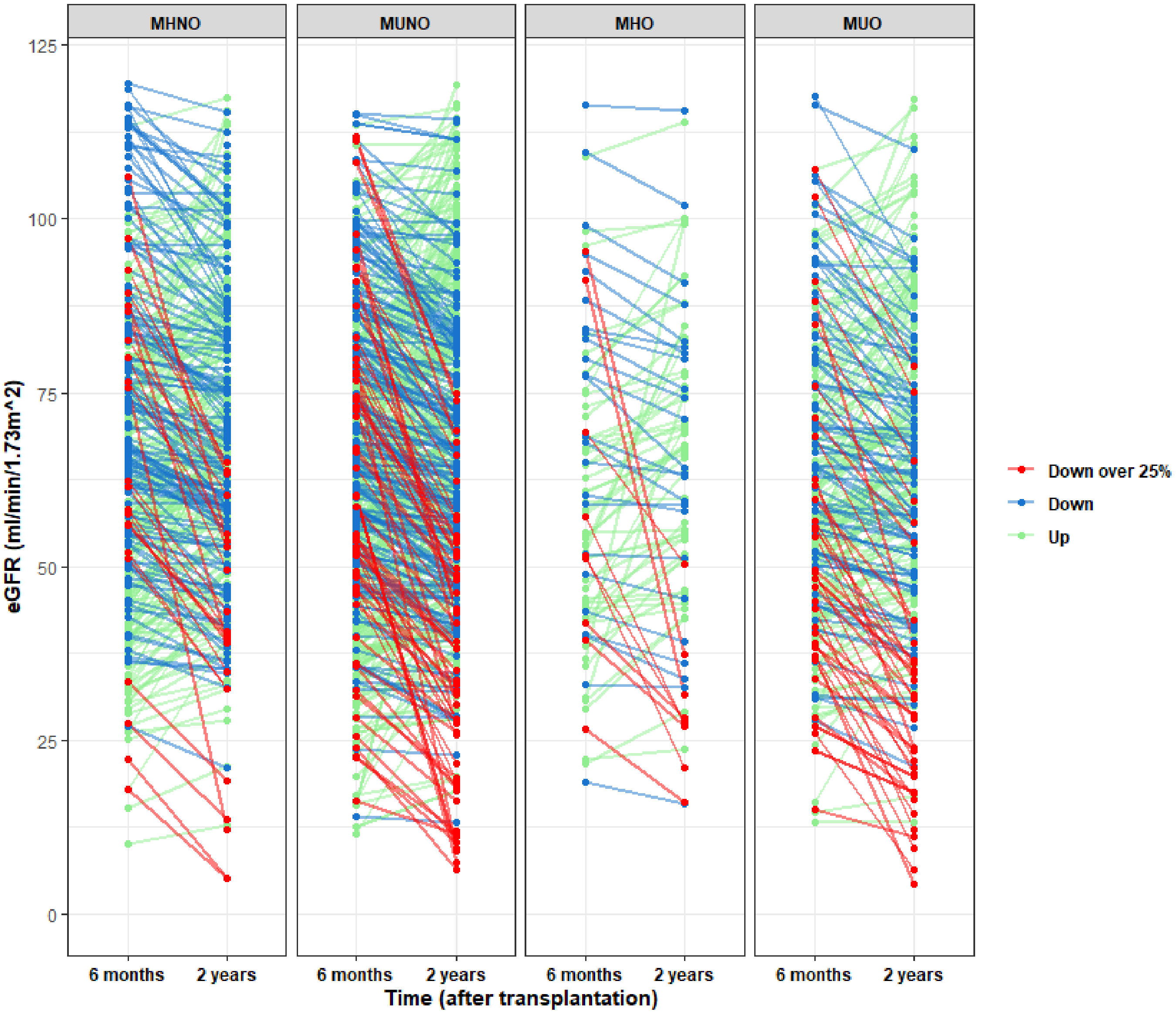

eGFR: estimated glomerular filtration rate; MUO: metabolically unhealthy overweight/obesity; MHO: metabolically healthy overweight/obesity; MUNO: metabolically unhealthy non-overweight/obesity; MHNO: metabolically healthy non-overweight/obesity

© 2023 Yan J et al. *JAMA Network Open.*

eFigure 3. Kaplan-Meier curve for eGFR decline among the metabolic phenotypes

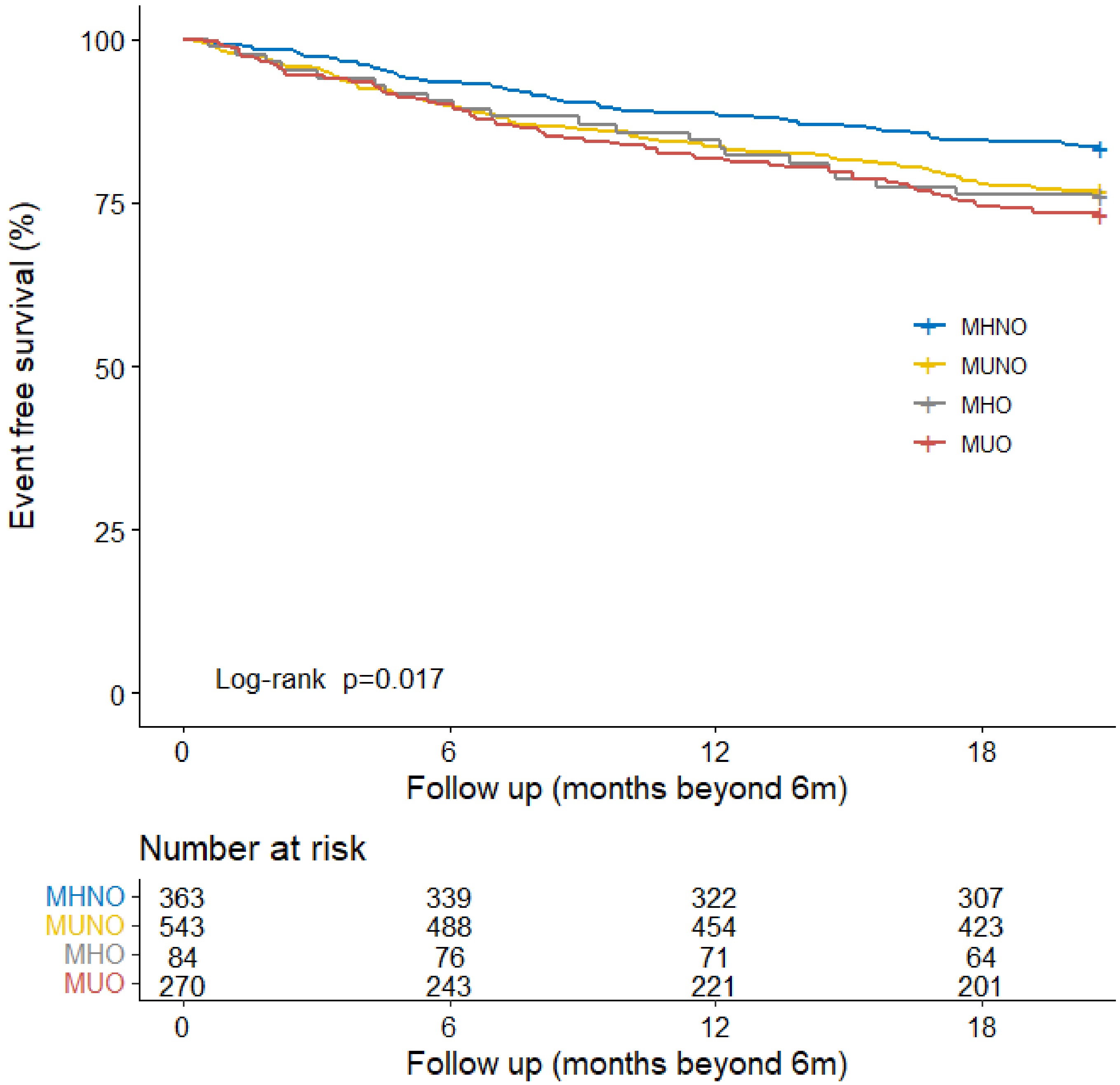

eGFR: estimated glomerular filtration rate; MUO: metabolically unhealthy overweight/obesity; MHO: metabolically healthy overweight/obesity; MUNO: metabolically unhealthy non-overweight/obesity; MHNO: metabolically healthy non-overweight/obesity

© 2023 Yan J et al. *JAMA Network Open.*

eFigure 4. Kaplan-Meier curve for overall graft survival among the metabolic phenotypes

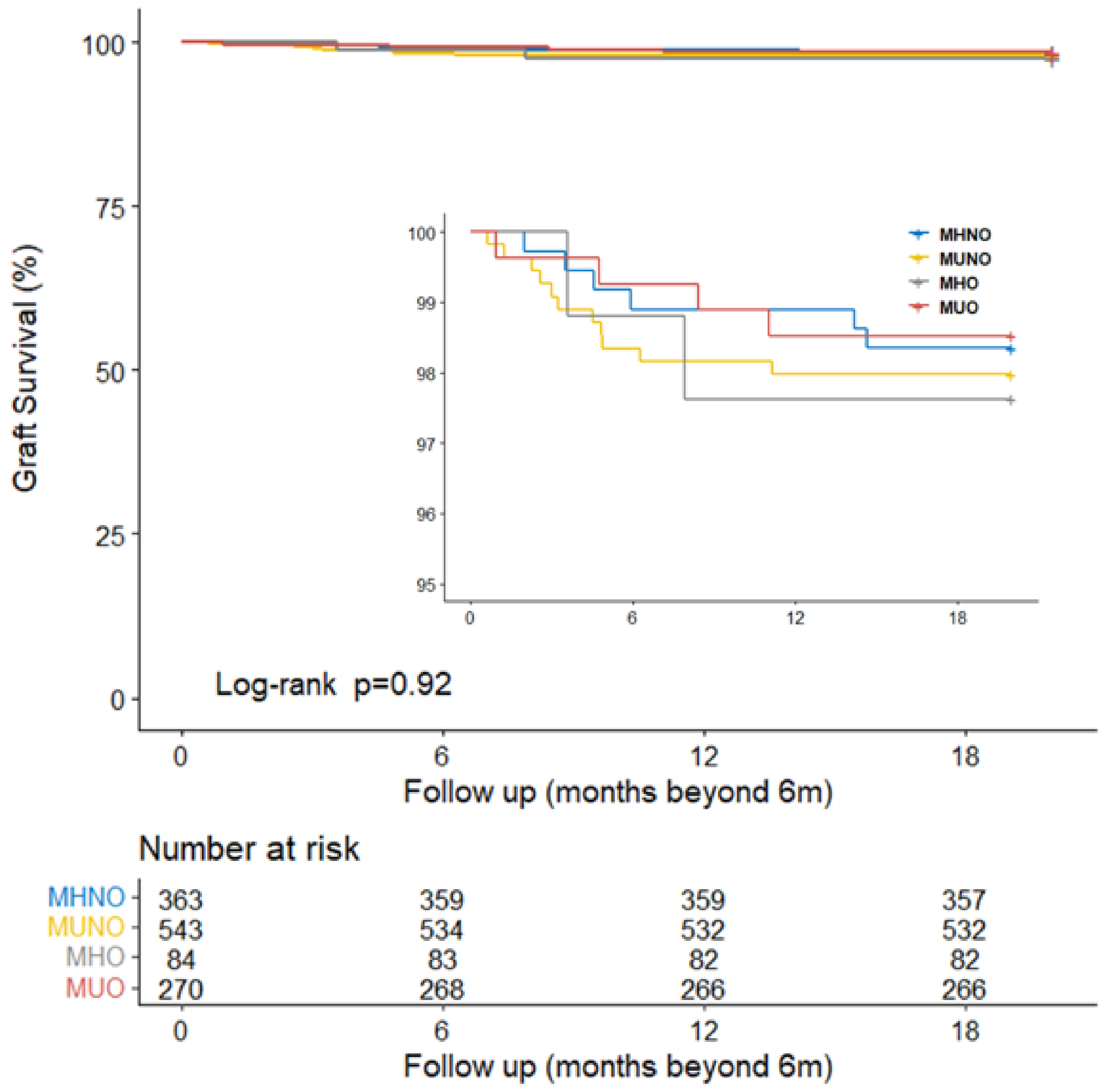

MUO: metabolically unhealthy overweight/obesity; MHO: metabolically healthy overweight/obesity; MUNO: metabolically unhealthy non-overweight/obesity; MHNO: metabolically healthy non-overweight/obesity

© 2023 Yan J et al. *JAMA Network Open.*

eReferences

1. Pan XF, Wang L, Pan A. Epidemiology and determinants of obesity in China. Lancet Diabetes Endocrinol. 2021;9(6):373-392.
2. Zhou BF, Cooperative Meta-Analysis Group of the Working Group on Obesity in China. Predictive values of body mass index and waist circumference for risk factors of certain related diseases in Chinese adults--study on optimal cut-off points of body mass index and waist circumference in Chinese adults. Biomed Environ Sci. 2002;15(1):83-96.
3. Expert Panel on Detection E, Treatment of High Blood Cholesterol in A. Executive Summary of The Third Report of The National Cholesterol Education Program (NCEP) Expert Panel on Detection, Evaluation, And Treatment of High Blood Cholesterol In Adults (Adult Treatment Panel III). *JAMA.* 2001;285(19):2486-2497.
4. Wang YC, Liang CS, Gopal DM, et al. Preclinical Systolic and Diastolic Dysfunctions in Metabolically Healthy and Unhealthy Obese Individuals. *Circ Heart Fail.* 2015;8(5):897-904.
5. Clayton PA, Lim WH, Wong G, Chadban SJ. Relationship between eGFR Decline and Hard Outcomes after Kidney Transplants. *J Am Soc Nephrol.* 2016;27(11):3440-3446.
6. Hilbrands L, Budde K, Bellini MI, et al. Allograft Function as Endpoint for Clinical Trials in Kidney Transplantation. *Transpl Int.* 2022;35:10139.
7. Levey AS, Coresh J, Greene T, et al. Using standardized serum creatinine values in the modification of diet in renal disease study equation for estimating glomerular filtration rate. *Ann Intern Med.* 2006;145(4):247-254.

© 2023 Yan J et al. *JAMA Network Open.*
