# Supplementary material for: Metabolic Risk Profile and Graft Function Deterioration 2 Years After Kidney Transplant
Source: JAMA Netw Open. 2023 Dec 27;6(12):e2349538. doi: 10.1001/jamanetworkopen.2023.49538 (PMC10753396; doi:10.1001/jamanetworkopen.2023.49538)
Supplement: Supplement 2. — Data Sharing Statement [file jamanetwopen-e2349538-s002.pdf]

# Data Sharing Statement

Yan. Metabolic Risk Profile and Graft Function Deterioration 2 Years After Kidney Transplant. *JAMA Netw Open*. Published December 27, 2023. doi:10.1001/jamanetworkopen.2023.49538

**Data**
**Data available:** Yes
**Data types:** Deidentified participant data
**How to access data:** monsoon585@foxmail.com
**When available:** With publication

**Supporting Documents**
**Document types:** None

**Additional Information**
**Who can access the data:** researchers whose proposed use of the data has been approved
**Types of analyses:** for research purpose only
**Mechanisms of data availability:** after approval of a proposal and with a signed data access agreement
